# Gamma irradiation-mediated inactivation of enveloped viruses with conservation of genome integrity: Potential application for SARS-CoV-2 inactivated vaccine development

**DOI:** 10.1515/biol-2021-0051

**Published:** 2021-06-02

**Authors:** Fouad A. Abolaban, Fathi M. Djouider

**Affiliations:** Nuclear Engineering Department, Faculty of Engineering, King Abdulaziz University, PO Box 80204, Jeddah, 21589, Saudi Arabia

**Keywords:** SARS-CoV-2 virus, virus genome, virus infectivity, gamma irradiation, virus inactivation, vaccine development

## Abstract

Radiation inactivation of enveloped viruses occurs as the result of damages at the molecular level of their genome. The rapidly emerging and ongoing coronavirus disease 2019 (COVID-19) pneumonia pandemic prompted by severe acute respiratory syndrome coronavirus 2 (SARS-CoV-2) is now a global health crisis and an economic devastation. The readiness of an active and safe vaccine against the COVID-19 has become a race against time in this unqualified global panic caused by this pandemic. In this review, which we hope will be helpful in the current situation of COVID-19, we analyze the potential use of γ-irradiation to inactivate this virus by damaging at the molecular level its genetic material. This inactivation is a vital step towards the design and development of an urgently needed, effective vaccine against this disease.

## 1 Introduction

Viruses (or virions) are subcellular particles, commonly spherical or rod-shaped, which composed of a protein capsid that contains their genetic material made of RNA or DNA. Sometimes the viral genome is protected by an additional outer envelope made of a lipid bilayer with spikes of glycoproteins inserted inside the viral envelope [1]. Viruses are classified based on their size, shape, envelope, and structure of their genome. Unlike bacteria, viruses lack cell organelles and thus have no metabolic activities on their own. To transcript and replicate, they entirely depend on the host biochemical machinery of eukaryotic or prokaryotic host cells [2]. Once inside the host cell, viruses can mutate through genome deletion, insertion, and/or substitution to novel strains of different virulence [3,4]. This viral mutation is the major obstacle for the development of new vaccines [5,6].

### 1.1 Key features of human coronavirus

Six human coronaviruses (HCoVs) were known before the COVID-19 outbreak: 229E and NL63 (alpha coronavirus), OC43, HKU1, SARS-CoV, and MERS-CoV (beta coronavirus) [7]. Severe acute respiratory syndrome (SARS) coronavirus (SARS-CoV) first emerged in South China in 2002–2003 to cause a large-scale epidemic with over 8,000 infections and more than 800 deaths [8]. The Middle East Respiratory Syndrome CoV (MERS-CoV) has caused a persistent epidemic in the Arabian Peninsula, especially in Saudi Arabia in 2012 [9]. SARS-CoV and MERS-CoV are enveloped positive-sense RNA viruses (size ranging from 70 to 90 nm) belonging to the Coronaviridae family. It was shown that rodents, avians, and mainly bats are reservoir host of these family viruses that can be potentially transmitted from animals to humans [10,11] due to the growing consumption of animal proteins including those from exotic wild mammals in China.

A novel strain of coronavirus, labeled as SARS-CoV-2 by the International Committee on Taxonomy of Viruses-Coronavirus Study Group [12], belonging to the beta-coronavirus lineage, shares around 80% identity to SARS-CoV [13,14]. This strain, believed to have started from a seafood market in the city of Wuhan, China, in December 2019, is now spreading and creating a chaos across the entire world [15,16]. Human-to-human transmission of this virus was officially confirmed on January 20th 2020 [10]. World Health Organization (WHO) declared this disease as a global pandemic on March 11, 2020 [17]. As of 29 January 2021, 27,901,760 cases are still active, 72,337,017 recovered, and 2,210,259 died in 235 countries or territories worldwide [18].

Similar to previous coronaviruses symptoms, SARS-CoV-2 is mainly affecting the lower respiratory track, ranging from mild respiratory disease to SARS and septic shock in advanced stages. Damages to the cardiovascular system, gut, kidneys, and brain have also been reported [13,19] along with vital organ failures in comorbid patients [20,21].

The genetic material of SARS-CoV-2 consists of single-stranded RNA backbone made of alternating 5-carbon sugar (ribose) and phosphate groups. Attached to this backbone are 29,891 to 29,903 adenine, uracil, cytosine, and guanine bases [22,23] encoding for 9,860 amino acids [10]. One third of the SARS-CoV-2 genome make up the four major structural proteins: spike glycoprotein (S), membrane protein (M), envelope protein (E), and nucleocapsid protein (N) accountable for some important functions in virus replication [24,25]. The remaining two‐thirds of its viral genome encode for 16 nonstructural proteins (nsp-1 to 16). Each of these nsps has a specific role in the life cycle of the virus and its pathogenicity [26,27]. For instance, nsp-1 is used by the virus to elude the host innate immune system [28], nsp-2 is indispensable for its replication, and nsp-9, in complex with nsp-8, is involved in RNA replication and virulence [29].

There is a 76.5% similarity in the amino acid sequences of the spike glycoprotein in SARS-CoV and SARS-CoV-2 [30]. SARS-CoV-2 seems to have greater binding affinity to the angiotensin converting enzyme 2 (ACE2) cell membrane receptor than the other SARS-CoV virus strains, suggesting a greater capacity of SARS-CoV-2 for human to human transmission [31,32]. There are speculations that cellular overexpression of human ACE2 (associated with the usage of medications such as ACE2 inhibitors, angiotensin II receptor blockers) could enhance the COVID-19 severity [21,33,34,35,36]. Among the four structural proteins of the SARS-CoV family, the spike glycoprotein S plays a key role in viral docking and cellular internalization [37]. It binds via the receptor-binding domain (RBD) in the S1 subunit to the ACE2 receptors [38,39], expressed especially on the plasma membrane of human respiratory epithelial host cells, and virtually in all other organs. The virus penetrates the host cell via endocytosis through its S2 subunit [40,41] and infects it by hijacking its molecular machinery to encode for RNA polymerase enzyme necessary for the replication of its own RNA genome [42,43]. Viral entry in cells is a critical phase in the course of the COVID-19 disease. Thus, inhibition of the viral binding and internalization in a host cell constitute a strategy for potential therapeutics against COVID-19 pandemic.

### 1.2 SARS-CoV-2 infectivity

Aerial transmission by expelled respiratory droplets is considered as the main direct transmission vector of the SARS-CoV-2 when in close contact with an infected person coughing, sneezing, or even talking [10,44,45,46]. Some findings have indicated that the virus may as well be airborne [31,47,48]. Indirect transmission may also occur via fomites when respiratory droplets from infected people land on object surfaces which can be touched by a receptive host [49,50].

The WHO recommends that SARS CoV-2 sample handling should be conducted in no less than a Biosafety Level 3 (BSL-3) laboratory using BSL-3 practices (WHO, 2020) [17,51]. The SARS-CoV-2 cytopathogenic effect is measured by its capability to infect a host cell. This is usually expressed by the tissue culture infectious dose, TCID_50/mL_, calculated using the method of Reed and Muench, [52], which is the viral titer at which 50% of the host cell lines are infected when inoculated *in vitro* with a diluted viral solution. However, because of some limitations with the *in vitro* tests (slow viral growth), the use of *in vivo* assays is taken after inoculated animals were sacrificed for within-host virus titering and pathological study [53].

Basic reproductive number {R}_{0}] is a key epidemiological factor used to measure the potential infectivity of virus-related outbreak [54,55]. It represents the average number of infected people caused by one infected individual during his/her whole contagious phase. Values of {R}_{0}] less than one means that the pandemic is most likely to stop propagating. In early stage of COVID-19 pandemic, realistic pooled values of {R}_{0}] were estimated in 29 studies, all done in China, using different mathematical methods [56]. The mean value of {R}_{0}] was evaluated as 3.38 ± 1.40 (95% confidence interval 1.9–6.49). {R}_{0}] was found to be between 2.43 to 3.10 in Italy in early stage of COVID-19 pandemic [54].

One of the key epidemiological factors in the COVID-19 pandemic is the incubation period, time elapsed between the exposure and the appearance of the first symptoms. Different pooled analysis of confirmed COVID-19 cases showed that the estimated incubation times were 5.1 days [57], 6.4 days (range 2.2–11.1) [58], and 5.0 days (range 2–14 days) [59]. Sanche and colleagues estimated, in late January 2020 in Mainland China, the average incubation period to 4.2 days. A time duration from symptoms onset to admission to hospital for treatment was estimated to 1.5 days in late January 2020 and the time from symptoms onset to death to 16.1 days [60].

Various *in vitro* studies showed that RNA viruses are less vulnerable to corruption due to their ability to promptly repair their genome damages by proof-reading, excision, and removing flawed RNA nucleotides that occur during their replication [61,62,63,64]. Exoribonuclease (ExoN) enzymes encoded by nsp-14 play a crucial role in maintaining the viral genome integrity [18,64,65,66]. Eckerle and workers reported that mutations in nsp-14 of SARS viruses lead to 15-fold increase in replication errors [67].

### 1.3 Vaccine strategy

To help prevent the spreading of the COVID-19 pandemic, most of the countries have adopted immediate measures consisting of global travel restrictions on movement, lockdowns, social distancing, patient self-isolation, and provision of medical care to infected people. Few curative methods using already known antiviral agents such as hydroxychloroquine and remdesivir were tested on patients [68,69,70]. However, results were not very encouraging for any of these agents to be considered as significant therapy yet. The best option for ending this pandemic and reestablishing a normal life remains, by far, the development of safe and effective prophylactic vaccines. This has triggered an extensive collaboration and a colossal mission between pharma companies and scientists to expedite vaccine development and production in less than a year instead of the normal 10-year period time. By the end of 2020, 259 COVID-19 vaccine projects were in the pipeline [71]. Frontrunning coronavirus vaccines, sharing the same purpose of stimulating the immune system against SARS-CoV-2, can be broadly categorized into three platforms:

- the classical inactivated virus vectored vaccines based on disrupting the viral genome through chemical or physical alterations. These viruses are no longer able to replicate to cause infection, but able to trigger an immune memory response [72].
- the full-length S glycoprotein- or RBD-based vaccines that generate target antigens in the infected cell [73].
- the groundbreaking DNA-, mRNA-based vaccines that encode in the host cell the full-length S glycoprotein as target antigen [74].

As of 29 January 2021, five vaccines went through the necessary multiple phases of trial to ensure safety, showing more than 90% efficacy. They have been approved and licensed for use by national and international public health regulators and are being rolled out worldwide (Table 1).

**Table 1:** COVID-19 vaccines currently available in the market (January 2021)

Vaccine	Platform	Inactivation method	Developer	Reference
BNT162b2	mRNA	—	Pfizer – BioNTech (USA, Germany)	[75,76]
mRNA-1273	mRNA	—	Moderna (USA)	[77,78]
AZD1222	Nonreplicating viral vector	Deletions in E1 and E3 genes in adenovirus vector to inhibit replications	University of Oxford AstraZeneca (UK)	[15,79,80]
CoronaVac	Inactivated SARS-CoV-2	β-Propiolactone to inhibit replication	Sinovac (China)	[29,81]
Sputnik V	Heterologous recombinant adenovirus (rAd26 and rAd5)	Deletions in E1 and E3 genes in adenovirus vector to inhibit replications	Gamaleya (Russia)	[82,83]

### 1.4 Different agents for SARS-CoV-2 inactivation

Virus inactivation for vaccine purposes was already known since the late 1800s [84]. In 1885, Pasteur laid the foundations of immunization with inactivated rabies virus cultured in rabbit spinal cords [85]. It was not until the discovery of the *in vitro* culture of viruses outside the host organism procedures that inactivated viral vaccine development was truly initiated. This allowed a large-scale production of viruses as source for inactivated vaccine purposes [86]. Vaccine producers are generally using virus growth on continuous cell lines to reduce production costs and increase vaccine safety. Once the virus has been purified, inactivation can be achieved using chemical or physical methods or a combination of the two. A wide range of chemical agents are used: ascorbic acid [87], derivatives of ethylenimine [88], and hydrogen peroxide [89]. However, formaldehyde [90] and β-propiolactone [91] are the most widely used for inactivation for decades. To avoid the extensive and time-consuming downstream processing to detoxify the virus cultures from chemical inactivators, the use of γ‐irradiation as a physical alternative method to chemical inactivation has been proposed by many authors. Preparation of experimental vaccines against several viral diseases using γ‐irradiation is reported in the literature: bluetongue [92], Venezuelan equine encephalitis [93], rabies [94], smallpox [95], influenza [96], HIV [97], Ebola [98], rotavirus [99], and polio [100].

The choice of an inactivation method preserving the viral epitope integrity is important since the damage of the envelop protein will lessen the efficacy of the vaccine [101]. Several studies showed that viral inactivation by formaldehyde, hydrogen peroxide, or binary ethylenimine derivatives is nonselective and can damage the envelope protein leading to a poor immune response [102,103]. Nevertheless, γ-irradiation has shown a superior inactivation method by preserving the viral antigens intact to trigger the immunogenicity while destroying nucleic acids to inhibit the viral replication in human cells [104,105]. This advantageous attribute of γ-irradiation can be ascribed to its high penetration depth that causes direct damage to nucleic acids without altering structural proteins [96,106,107].

Due to the potentially dangerous consequences of SARS-CoV-2 human infection, extreme attention should be paid to ensure that inactivation procedures are efficient. Effective inactivation of the SARS-CoV-2 is vital as it allows research, especially the development of new vaccines, to be conducted under safe conditions [108]. Various methods are already available for SARS-CoV effective inactivation [109] and could be tested on SARS-CoV-2 since these two viruses share a great deal of genome: ultraviolet radiation, thermal treatment, extreme pH values, and commonly used disinfectants offer an effective virus inactivation. Duan and colleagues reported an inactivation of SARS-CoV virus after 15 min exposure to ultraviolet C, whereas ultraviolet A and B had no effect on its viability, irrespective of the duration of the exposure [110]. Heat can denature SARS-CoV secondary structural proteins. A complete inactivation of this virus at 75°C for 45 min was reported by Darnell and colleagues [111]. For inactivation with detergents, Gerlach et al. showed that SARS-CoV-2 can be efficiently inactivated by 70% ethanol, 0.1% hydrogen peroxide, and 0.1% sodium sulphate, commonly available in hand soaps, within 60 s of exposure on various surfaces [112]. pH has a great effect on the viability of SARS-CoV-2. Chan et al. reported that the virus survived for up to 6 days in a medium with pH range [5–9], but lost between 2.9 and 5.33 log_10_ infectivity. At pH 4 and pH 11, it remained viable for 1–2 days. At extreme pHs (pH 2–3 and pH 11–12), the virus lost 5.25 log_10_ infectivity within only 1 day [16].

**Figure 1:**
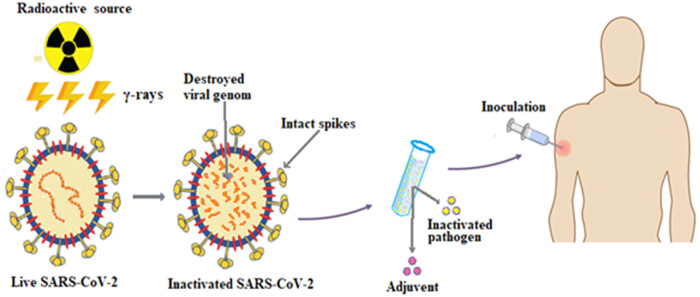
Schematic diagram showing the development of SARS-CoV-2 vaccine using radiation-induced inactivation of live virus.

In this perspective, γ-inactivation of viruses could be an important and promising tool for SARS-CoV-2 vaccine development (Figure 1). In this review, we analyze the potential use of γ-irradiation to inactivate the SARS-CoV-2 by altering its genetic material while preserving its structural proteins.

### 1.5 Radiolysis of water in biological matter: mechanisms of radiobiological action

The two major processes in the interaction of energetic photons or charged particles with aqueous biological medium are ionization and excitation of water molecules and biologically important macromolecules such as DNA, RNA, lipids, and proteins [113,114]. The electrons liberated in the ionization have enough energy to ionize further molecules in a manner that energy is deposited in spurs. When their energy falls below the ionization threshold of water, the electrons become solvated {e}_{aq}^{-}]. Excited water molecules can dissociate into free radicals {\text{OH}}^{\bullet }] and {\text{H}}^{\bullet }]. These physicochemical processes, concentrated in tracks along the path of the ionizing species, are complete approximately 10^−12^ s after the absorption of the ionizing radiation [115,116]. The radiolysis products either react with each other within the spurs to produce reactive oxygen species (ROS), such as superoxide radical O_2_^•−^ and its conjugate perhydroxyl radical HO_2_^•^, or diffuse homogeneously into the bulk of the biological medium where they are scavenged by biological macromolecules. The spur diffusion is complete approximately 10^−7^ s after the absorption of the ionizing radiation [117].

### 1.6 Mechanism of viral inactivation

γ-irradiation disrupts viruses by altering mainly their RNA genetic material. The number of nitrogenous bases and their sequence in the RNA is crucial for determining the viral sensitivity towards γ-irradiation. The more target nucleotides, the more likely the nucleic acid genome will be damaged for a given absorbed dose [118]. The mechanism behind this damage falls into two types: direct and indirect. Direct damage is caused by the radiation-induced cleavage of the sugar-phosphate backbone or the cross-linking, deletion, substitution, and insertion in the sequence of nitrogen bases [119,120]. Indirect damage is attributed to the oxidative stress of the radiolytically produced ROS on the viral material, leading to its fragmentation and cross-linking [121,122]. A minimum energy deposition of 17.5 eV within a critical distance of 6 Å from the nucleotide (corresponding to a sensitive spherical volume of 0.596 nm^3^) can induce a lethal damage to the viral RNA [123]. Disruption to the protein capsid and the lipid bilayer envelope, by lipid and protein peroxidation chain reactions, may as well result in the reduction of viral pathogenicity [70,124,125]. It has been reported that, for viruses belonging to the enveloped Coronaviridae family, the conformational changes in the spike glycoprotein S block the viral binding to the host cell plasma membrane and prevent cellular internalization, the first stage in viral infection [111,126]. Studies suggested that genetic material rather than protein and lipid envelopes is likely to be the primary target for viral inactivation [107,127]. Nims et al. reported that the presence or absence of a viral envelope does not seem to be a major factor of inactivation by γ-irradiation [128].

Single-strand break (for single-stranded viruses) and double-strand break (for double-stranded viruses) are generally sufficient to inactivate the viral genome [129]. Based on the hypothesis of the single-hit-single-target (SHST) model [130,131], the inactivation of viruses is typically expressed by the following relationship [132]:

(1) N(D)=\text{ }{N}_{0\text{ }}{10}^{-\frac{D}{{D}_{10}}}]

where {N}_{0}]and N(D)] are the virus population before and after the irradiation, respectively, D] being the radiation dose. {D}_{10}] depicts the required irradiation dose to reduce the initial virus titer by 90% (or reduce the population by a factor of 10). {D}_{10}] values vary between different types of viruses mainly due to the significant differences in their genome, capsid morphology, and the presence or absence of an envelope. For convenience, the viral inactivation is expressed in terms of log_10_ reduction, which is the logarithm base 10 of the ratio of the viral titer before ({N}_{0})] and after (N(D))] the inactivation:

(2) {\text{log}}_{10}\hspace{.5em}\text{reduction}=\hspace{.25em}{\text{log}}_{10}\left(\frac{{N}_{0}}{N(D)}\right)]

It should be noted that {D}_{10}\hspace{.25em}]corresponds to 1\hspace{.25em}{\text{log}}_{10}]. For instance, 1\hspace{.25em}{\text{log}}_{10}\hspace{.5em}\text{reduction}] corresponds to 90% reduction (or 10-fold) and 2\hspace{.25em}{\text{log}}_{10}\hspace{.5em}\text{reduction}] corresponds to 99% reduction (or 100-fold).

Feldmann and colleagues showed that the inactivation was inversely correlated with genome size [133]. They measured the radiation doses for a 6 log_10_ reduction and found 2 Mrads for coronaviruses (∼29 kb genome size), 4 Mrads for filoviruses (∼19 kb), 8 Mrads for arenaviruses, bunyaviruses, orthomyxoviruses, and paramyxoviruses (∼13 kb) and 10 Mrads for flaviviruses (∼9 kb). Viruses having single-strand nucleic acid present the highest radiosensitivity. Hume and colleagues [127] reported that the three enveloped single-stranded RNA viruses of similar sizes, namely morbillivirus (90–150 nm), bunyavirus (90–120 nm), and rhabdovirus (70–150 nm), showed a comparable *D*_10_ values (2.53, 2.61, and 2.71 kGy respectively) when irradiated under the same experimental protocol.

Leung and workers cultured SARS-CoV-2 on Vero cells in the presence of 1% fetal bovine serum and 1% l-glutamine. Virus-containing supernatants were titered after irradiation [134]. The *D*_10_ was 1.6 kGy and the complete inactivation of the SARS-CoV-2 was attained with an absorbed dose of 10 kGy, value lower than the 20 kGy previously reported value for the similar SARS-CoV [133]. Even though the single-stranded RNA viruses may present a certain radioresistance, Nims and colleagues showed that no strong clue can explain the discrepancies in the log_10_ values for virus inactivation by γ-irradiation, as shown in Table 2 [135]. Multiple causative parameters may be involved in this discrepancy, including, but not limited to, sera matrices preparation variability from sample-to-sample, variability in γ-irradiation procedures (dose rate), and variability in the purity of the virus stock quality.

**Table 2:** Viral properties of some enveloped virus families. Efficacy of γ-irradiation on the log_10_ reduction

Virus	Family	Morphology (not to scale)	Size (nm)	Genome size (kb)*	Nucleic acid genome	*D*_10_ (kGy)	log_10_ reduction/kGy**c**	References
IBR^b^	Herpesviridae	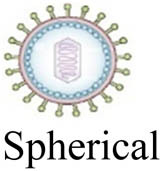	100–120	120–230	Double-stranded DNA	3.22	0.310	[136,137]
APV	Poxviridae	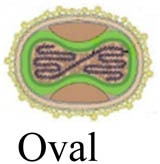	240–300	130–260	Double-stranded DNA	2.20	0.456	[138,139]
PI3^b^	Paramyxoviridae	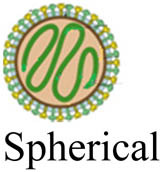	100–200	13–19	Single-stranded RNA	4.78	0.209	[128,140]
BVDV^a^	Flaviviridae	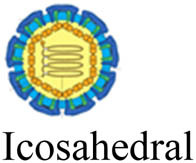	50–70	9–13	Single-stranded RNA	5.05	0.198	[141,142]
SARS-CoV-2	Coronaviridae	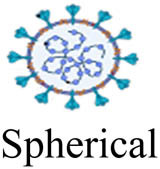	20–25	26–32	Single-stranded RNA	1.60	0.625	[134,143]
BEFV^a^	Rhabdoviridae	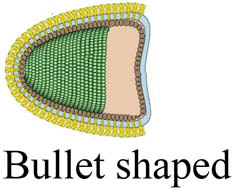	75 × 150	10–16	Single-stranded RNA	2.94	0.340	[142,144]
Akabane^a^	Bunyavuridae	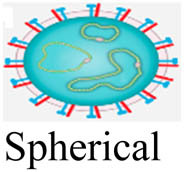	80–120	11–23	Single-stranded RNA	2.50	0.400	[142,145]
Aino^a^	Bunyavuridae	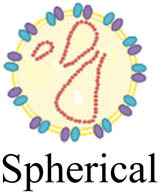	80–120	11–23	Single-stranded RNA	3.45	0.290	[142,146]

APV: avian poxvirus, PI3: parainfluenza virus type 3, IBR: infectious bovine rhinotracheitis, BVDV: bovine viral diarrhea virus, BEFV: bovine ephemeral fever virus.

* A kilobase (kb) pair is a unit of genome size measurement equal to 1,000 base pairs of DNA or RNA.

a Viruses in bovine serum.

b Viruses spiked into fetal bovine serum.

c log_10_ reduction per kGy represents the slope of the viral inactivation (log_10_ reduction in titer) vs radiation dose in kGy.

In another study, Schmidt and workers [132] investigated the fractionated and continuous electron beam irradiation of four different types of viruses: the Human Immunodeficiency Virus-2 (Retroviridae enveloped HIV-2), the Hepatovirus A (Picornaviridae non-enveloped HAV), the Pseudorabies Virus (Herpesviridae, enveloped PRV), and the Porcine Parvovirus (Parvoviridae non-enveloped PPV). The cell lines for these viruses were respectively human T lymphocyte cells, mink-lung cells, embryonal rhesus monkey kidney cells, and pig kidney cells. The irradiation doses for the continuous beam were multiple of 3.4 kGy and up to 34 kGy, while for the fractionated beam, a dose of 3.4 Gy was applied up to 10 times. The {D}_{10}] values were estimated from the regression curve TCID_50/mL_ vs absorbed dose. Except for PRV, each type of virus showed a slightly greater radioresistance in continuous than fractionated irradiation. This might be explained by the ability of the viruses to repair their sublethal damages to their viral genome between two successive fractions. The least resistant was the PRV (continuous beam: 5.6 kGy, fractionated beam: 5.8 kGy) and the most resistant was the HIV-2 (continuous beam: 9 kGy, fractionated beam: 8 kGy).

The presence of solutes in the irradiation culture of viruses renders the radiation-induced inactivation of viruses less efficient. de Roda Husman et al. [125] compared the inactivation of the respiratory feline calicivirus (FeCV) and the enteric canine calicivirus (CaCV) with the Escherichia bacteriophage MS2 in viral culture of different protein concentration. They reported a 3\hspace{.25em}{\text{log}}_{10}] reduction factor at a dose of ∼120 Gy when γ-irradiating MS2 in tap water or in low-protein-contents. They obtained doses of 500 and 300 Gy, for a 3 log_10_ reductions factors, for FeCV and CaCV, respectively, in low-protein-content cultures. No or very little inactivation was observed when MS2, FeCV, and CaCV were γ-irradiated in high-protein-content culture. The presence of OH˙ and H˙ scavengers, such as proteins, lipids, etc., significantly reduces the viral inactivation. The authors also reported that FeCV and CaCV showed a second-order kinetics, with faster inactivation happening at doses between 0 and 400 Gy and slower inactivation happening at doses from 400 to 800 Gy.

## 2 Conclusion

The eruption of the SARS-CoV-2 new virus poses a real challenge and worries in a sense that its attributes are initially unknown and very limited viral data on COVID-19 infection is currently available. The virus is still infecting populations globally. Previous studies from other viruses and particularly the SARS-CoV and MERS-CoV have showed that damaging the genetic material can destroy infectivity while retaining immunogenicity. This review showed that ionizing radiation can potentially inactivate almost all the RNA viruses, by disrupting their genomic material. This could suggest a basis for developing a potential γ-inactivated virus-based vaccine against the spread of the COVID-19 pandemic. Improving its immunogenicity and preventing any potential undesired effects that could compromise the safety of the vaccine are of course other important factors which should also be taken into full consideration and thoroughly examined.

## References

[1] Mäntynen S, Sundberg L-R, Oksanen H, Poranen M. Half a century of research on membrane-containing bacteriophages: bringing new concepts to modern virology. Viruses [Internet]. 2019 Jan 18;11(1):76. Available from: http://www.mdpi.com/1999-4915/11/1/76 10.3390/v11010076 PMC6356626 30669250

[2] Menéndez-Arias L, Andino R. Viral polymerases. Virus Res [Internet]. 2017 Apr;234:1–3. Available from: https://linkinghub.elsevier.com/retrieve/pii/S0168170217301284 10.1016/j.virusres.2017.02.003 PMC5710837 28188807

[3] Islam MR, Hoque MN, Rahman MS, Alam ASMRU, Akther M, Puspo JA, et al. Genome-wide analysis of SARS-CoV-2 virus strains circulating worldwide implicates heterogeneity. Sci Rep [Internet]. 2020 Dec 1 [cited 2021 Mar 11];10(1):14004. Available from: https://doi.org/10.1038/s41598-020-70812-6

[4] Pachetti M, Marini B, Benedetti F, Giudici F, Mauro E, Storici P, et al. Emerging SARS-CoV-2 mutation hot spots include a novel RNA-dependent-RNA polymerase variant. J Transl Med [Internet]. 2020 Apr 22 [cited 2021 Mar 11];18(1):1–9. Available from: https://pubmed.ncbi.nlm.nih.gov/32321524/10.1186/s12967-020-02344-6 PMC7174922 32321524

[5] Servín-Blanco R, Zamora-Alvarado R, Gevorkian G, Manoutcharian K. Antigenic variability: obstacles on the road to vaccines against traditionally difficult targets. Hum Vaccin Immunother [Internet]. 2016 Oct 2;12(10):2640–8. Available from: https://www.tandfonline.com/doi/full/10.1080/21645515.2016.1191718 10.1080/21645515.2016.1191718 PMC5085008 27295540

[6] Carlson JM, Le AQ, Shahid A, Brumme ZL. HIV-1 adaptation to HLA: a window into virus–host immune interactions. Trends Microbiol [Internet]. 2015 Apr;23(4):212–24. Available from: https://linkinghub.elsevier.com/retrieve/pii/S0966842X14002650 10.1016/j.tim.2014.12.008 25613992

[7] Ksiazek TG, Erdman D, Goldsmith CS, Zaki SR, Peret T, Emery S, et al. A novel coronavirus associated with severe acute respiratory syndrome. N Engl J Med. 2003;348(20):1953–66. 10.1056/NEJMoa030781 12690092

[8] Cheng VCC, Lau SKP, Woo PCY, Yuen Y. Severe acute respiratory syndrome coronavirus as an agent of emerging and reemerging infection. Clin Microbiol Rev [Internet]. 2007 [cited 2020 Aug 9];20(4):660–94. Available from: http://cmr.asm.org/10.1128/CMR.00023-07 PMC2176051 17934078

[9] Zaki AM, van Boheemen S, Bestebroer TM, Osterhaus ADME, Fouchier RAM. Isolation of a novel coronavirus from a man with pneumonia in Saudi Arabia. N Engl J Med [Internet]. 2012 Nov 8;367(19):1814–20. Available from: http://www.nejm.org/doi/abs/10.1056/NEJMoa1211721 10.1056/NEJMoa1211721 23075143

[10] Chan JFW, Yuan S, Kok KH, To KKW, Chu H, Yang J, et al. A familial cluster of pneumonia associated with the 2019 novel coronavirus indicating person-to-person transmission: a study of a family cluster. Lancet [Internet]. 2020 Feb;39;5(10223):514–23. Available from: https://linkinghub.elsevier.com/retrieve/pii/S0140673620301549 10.1016/S0140-6736(20)30154-9 PMC7159286 31986261

[11] Lun Z-R, Qu L-H. Animal-to-human SARS-associated coronavirus transmission? Emerg Infect Dis [Internet]. 2004 May;10(5):959. Available from: http://wwwnc.cdc.gov/eid/article/10/5/04-0022_article.htm 10.3201/eid1005.040022 PMC3323218 15216845

[12] Gorbalenya AE, Baker SC, Baric RS, de Groot RJ, Drosten C, Gulyaeva AA, et al. Severe acute respiratory syndrome-related coronavirus: the species and its viruses-a statement of the coronavirus study group; 2020 [cited 2020 Aug 12];1:1–20. Available from: https://doi.org/10.1101/2020.02.07.937862

[13] Huang C, Wang Y, Li X, Ren L, Zhao J, Hu Y, et al. Clinical features of patients infected with 2019 novel coronavirus in Wuhan, China. Lancet. 2020;395(10223):497–506. 10.1016/S0140-6736(20)30183-5 PMC7159299 31986264

[14] Rabaan AA, Al-Ahmed SH, Haque S, Sah R, Tiwari R, Malik YS, et al. SARS-CoV-2, SARS-CoV, and MERS-CoV: a comparative overview. Infez Med. 2020;28(2):174–84. 32275259

[15] Ramasamy MN, Minassian AM, Ewer KJ, Flaxman AL, Folegatti PM, Owens DR, et al. Safety and immunogenicity of ChAdOx1 nCoV-19 vaccine administered in a prime-boost regimen in young and old adults (COV002): a single-blind, randomised, controlled, phase 2/3 trial. Lancet [Internet]. 2020 Dec 19 [cited 2021 Feb 11];396(10267):1979–93. 10.1016/S0140-6736(20)32466-1 PMC7674972 33220855

[16] Chan KH, Sridhar S, Zhang RR, Chu H, Fung AYF, Chan G, et al. Factors affecting stability and infectivity of SARS-CoV-2. J Hosp Infect. 2020 Oct 1;106(2):226–31. 10.1016/j.jhin.2020.07.009 PMC7343644 32652214

[17] WHO. WHO coronavirus disease (COVID-19) dashboard. Geneva: World Health Organization; 2020.

[18] Ojha R, Gupta N, Naik B, Singh S, Verma VK, Prusty D, et al. High throughput and comprehensive approach to develop multiepitope vaccine against minacious COVID-19. Eur J Pharm Sci [Internet]. 2020 Aug 1 [cited 2021 Feb 11];151:105375. Available from: https://www.ncbi.nlm.nih.gov/pmc/articles/PMC7224663/10.1016/j.ejps.2020.105375 PMC7224663 32417398

[19] Shi S, Qin M, Shen B, Cai Y, Liu T, Yang F, et al. Association of cardiac injury with mortality in hospitalized patients with COVID-19 in Wuhan, China Supplemental content. JAMA Cardiol [Internet]. 2020 [cited 2020 Aug 9];5(7):802–10. Available from: https://jamanetwork.com/10.1001/jamacardio.2020.0950 PMC7097841 32211816

[20] Sanyaolu A, Okorie C, Marinkovic A, Patidar R, Younis K, Desai P, et al. Comorbidity and its impact on patients with COVID-19. SN Compr Clin Med [Internet]. 2020 Aug 25;2(8):1069–76. Available from: http://link.springer.com/10.1007/s42399-020-00363-4 10.1007/s42399-020-00363-4 PMC7314621 32838147

[21] Yang J, Zheng Y, Gou X, Pu K, Chen Z, Guo Q, et al. Prevalence of comorbidities in the novel Wuhan coronavirus (COVID-19) infection: a systematic review and meta-analysis. Int J Infect Dis [Internet]; 2020 [cited 2020 Aug 9]. Available from: https://www.sciencedirect.com/science/article/pii/S1201971220301363

[22] Hassan SA, Sheikh FN, Jamal S, Ezeh JK, Akhtar A. Coronavirus (COVID-19): a review of clinical features, diagnosis, and treatment. Cureus. 2020 Mar 21;12:1–7. 10.7759/cureus.7355 PMC7170025 32328367

[23] Wu F, Zhao S, Yu B, Chen YM, Wang W, Song ZG, et al. A new coronavirus associated with human respiratory disease in China. Nature. 2020 Mar 12;579(7798):265–9. 10.1038/s41586-020-2008-3 PMC7094943 32015508

[24] Luan J, Lu Y, Jin X. Research LZ-B and biophysical, 2020 U. Spike protein recognition of mammalian ACE2 predicts the host range and an optimized ACE2 for SARS-CoV-2 infection. Biochem Biophys Res Commun. 2020 May 21;526(1):165–9. 10.1016/j.bbrc.2020.03.047 PMC7102515 32201080

[25] Tortorici MA, Veesler D. Structural insights into coronavirus entry. Adv Virus Res. 2019;105:93–116. 10.1016/bs.aivir.2019.08.002. PMC7112261 31522710

[26] Raj R. Analysis of non-structural proteins, NSPs of SARS-CoV-2 as targets for computational drug designing. Biochem Biophys Rep [Internet]. 2021 Mar [cited 2021 Mar 11];25:100847. Available from: https://www.ncbi.nlm.nih.gov/pmc/articles/PMC7750489/10.1016/j.bbrep.2020.100847 PMC7750489 33364445

[27] Silva SJR, Alves da Silva CT, Mendes RPG, Pena L. Role of nonstructural proteins in the pathogenesis of SARS‐CoV‐2. J Med Virol [Internet]. 2020 Sep 2 [cited 2021 Mar 11];92(9):1427–9. Available from: https://onlinelibrary.wiley.com/doi/abs/10.1002/jmv.25858 10.1002/jmv.25858 PMC7262198 32270884

[28] Clark LK, Green TJ, Petit CM. Structure of nonstructural protein 1 from SARS-CoV-2. J Virol [Internet]. 2020 Nov 24 [cited 2021 Mar 11];95(4):1–12. Available from: http://jvi.asm.org/10.1128/JVI.02019-20 PMC7851544 33234675

[29] Gao Y, Yan L, Huang Y, Liu F, Zhao Y, Cao L, et al. Structure of the RNA-dependent RNA polymerase from COVID-19 virus. Science (80) [Internet]. 2020 May 15 [cited 2021 Mar 11];368(6492):779–82. Available from: https://pubmed.ncbi.nlm.nih.gov/32277040/10.1126/science.abb7498 PMC7164392 32277040

[30] Xu X, Chen P, Wang J, Feng J, Zhou H, Li X, et al. Evolution of the novel coronavirus from the ongoing Wuhan outbreak and modeling of its spike protein for risk of human transmission. Sci China Life Sci [Internet]. 2020 Mar 21;63(3):457–60. Available from: http://link.springer.com/10.1007/s11427-020-1637-5 10.1007/s11427-020-1637-5 PMC7089049 32009228

[31] Yao M, Zhang L, Ma J, Zhou L. On airborne transmission and control of SARS-Cov-2. Sci Total Environ [Internet]. 2020 Aug;731:139178. Available from: https://linkinghub.elsevier.com/retrieve/pii/S0048969720326954 10.1016/j.scitotenv.2020.139178 PMC7198171 32388162

[32] Wan Y, Shang J, Graham R, Baric RS, Li F. Receptor recognition by the novel coronavirus from Wuhan: an analysis based on decade-long structural studies of SARS coronavirus. In: Gallagher T, editor. J Virol [Internet]. 2020 Jan 29;94(7):1–25. Available from: https://jvi.asm.org/content/94/7/e00127-20 10.1128/JVI.00127-20 PMC7081895 31996437

[33] Yang X-H, Deng W, Tong Z, Liu Y-X, Zhang L-F, Zhu H, et al. Comparative medicine mice transgenic for human angiotensin-converting enzyme 2 provide a model for SARS coronavirus Infection [Internet]. ingentaconnect.com; 2007 [cited 2020 Aug 9]. Available from: https://www.ingentaconnect.com/content/aalas/cm/2007/00000057/00000005/art00003 17974127

[34] Devaux CA, Rolain J-M, Raoult D. ACE2 receptor polymorphism: Susceptibility to SARS-CoV-2, hypertension, multi-organ failure, and COVID-19 disease outcome. J Microbiol Immunol Infect [Internet]. 2020 Jun;53(3):425–35. Available from: https://linkinghub.elsevier.com/retrieve/pii/S1684118220301092 10.1016/j.jmii.2020.04.015 PMC7201239 32414646

[35] Li Q, Guan X, Wu P, Wang X, Zhou L, Tong Y, et al. Early transmission dynamics in Wuhan, China, of novel coronavirus–infected pneumonia. Mass Med Soc [Internet]. [cited 2020 Aug 9];382:1199–207. Available from: https://www.nejm.org/doi/full/10.1056/NEJMOa2001316 10.1056/NEJMoa2001316 PMC7121484 31995857

[36] Li Q, Guan X, Wu P, Wang X, Zhou L, Tong Y, et al. Early transmission dynamics in Wuhan, China, of novel coronavirus-infected pneumonia. New Engl J Med. 2020;382:1199–207. 10.1056/NEJMoa2001316 PMC7121484 31995857

[37] Tai W, He L, Zhang X, Pu J, Voronin D, Jiang S, et al. Characterization of the receptor-binding domain (RBD) of 2019 novel coronavirus: implication for development of RBD protein as a viral attachment inhibitor. naturecom [Internet]. 2020 [cited 2020 Aug 12]. Available from: https://www.nature.com/articles/s41423-020-0400-4?report=reader 10.1038/s41423-020-0400-4 PMC7091888 32203189

[38] Shang J, Ye G, Shi K, Wan Y, Luo C, Aihara H, et al. Structural basis of receptor recognition by SARS-CoV-2. Nature. 2020 May 14;581(7807):221–4. 10.1038/s41586-020-2179-y PMC7328981 32225175

[39] Verdecchia P, Cavallini C, Spanevello A, Angeli F. The pivotal link between ACE2 deficiency and SARS-CoV-2 infection. Eur J Int Med. 2020;76:14–20. 10.1016/j.ejim.2020.04.037 PMC7167588 32336612

[40] Grove J, Marsh M. The cell biology of receptor-mediated virus entry. J Cell Biol. 2011;195:1071–82. 10.1083/jcb.201108131 PMC3246895 22123832

[41] Walls AC, Park YJ, Tortorici MA, Wall A, McGuire AT, Veesler D. Structure, function, and antigenicity of the SARS-CoV-2 spike glycoprotein. Cell. 2020 Apr 16;181(2):281–92. 10.1016/j.cell.2020.02.058 PMC7102599 32155444

[42] Sullivan CS, Ganem D. microRNAs and viral infection. Mol Cell. 2005;20(1):3–7. 10.1016/j.molcel.2005.09.012 16209940

[43] South AM, Diz DI, Chappell MC. COVID-19, ACE2, and the cardiovascular consequences. Am J Physiol Circ Physiol. 2020;318:1084–90. 10.1152/ajpheart.00217.2020 PMC7191628 32228252

[44] Liu J, Liao X, Qian S, Yuan J, Wang F, Liu Y, et al. Community transmission of severe acute respiratory syndrome coronavirus 2, Shenzhen, China, 2020. Emerg Infect Dis. 2020 Jun;26(6):1320–23. 10.3201/eid2606.200239. PMC7258448 32125269

[45] Ghinai I, McPherson TD, Hunter JC, Kirking HL, Christiansen D, Joshi K, et al. First known person-to-person transmission of severe acute respiratory syndrome coronavirus 2 (SARS-CoV-2) in the USA. Lancet. 2020;395:1137–44. 10.1016/S0140-6736(20)30607-3 PMC7158585 32178768

[46] Lu J, Gu J, Li K, Xu C, Su W, Lai Z, et al. Early release-COVID-19 outbreak associated with air conditioning in restaurant, Guangzhou, China, 2020; 2020. 10.3201/eid2607.200764 PMC7323555 32240078

[47] Frontera A, Martin C, Vlachos K, Sgubin G. Regional air pollution persistence links to covid19 infection zoning. J Infect. 2020;81:318–56. 10.1016/j.jinf.2020.03.045 PMC7151372 32283151

[48] Morawska L, Cao J. Airborne transmission of SARS-CoV-2: the world should face the reality. Environ Int [Internet]. 2020 Jun;139:105730. Available from: https://linkinghub.elsevier.com/retrieve/pii/S016041202031254X 10.1016/j.envint.2020.105730 PMC7151430 32294574

[49] Chia PY, Coleman KK, Tan YK, Ong SWX, Gum M, Lau SK, et al. Detection of air and surface contamination by SARS-CoV-2 in hospital rooms of infected patients. Nat Commun [Internet]. 2020 Dec 29;11(1):2800. Available from: http://www.nature.com/articles/s41467-020-16670-2 10.1038/s41467-020-16670-2 PMC7260225 32472043

[50] Guo Z-D, Wang Z-Y, Zhang S-F, Li X, Li L, Li C, et al. Aerosol and surface distribution of severe acute respiratory syndrome coronavirus 2 in hospital wards, Wuhan, China, 2020. Emerg Infect Dis. 2020;26(7):10–3201. 10.3201/eid2607.200885 PMC7323510 32275497

[51] WHO. WHO-I guidance. Laboratory biosafety guidance related to the novel coronavirus (2019-nCoV). Geneva: World Health Organization; 2020.

[52] Ramakrishnan MA. Determination of 50% endpoint titer using a simple formula. Artic World J Virol [Internet]. 2016 [cited 2020 Aug 12];5(2):85. Available from: http://dx.doi.org/10.5501/wjv.v5.i2.85 10.5501/wjv.v5.i2.85 PMC4861875 27175354

[53] Gombold J, Karakasidis S, Niksa P, Podczasy J, Neumann K, Richardson J, et al. Systematic evaluation of in vitro and in vivo adventitious virus assays for the detection of viral contamination of cell banks and biological products. Vaccine [Internet]. 2014 May 19 [cited 2021 Mar 11];32(24):2916–26. Available from: https://pubmed.ncbi.nlm.nih.gov/24681273/10.1016/j.vaccine.2014.02.021 PMC4526145 24681273

[54] D’Arienzo M, Coniglio A. Assessment of the SARS-CoV-2 basic reproduction number, R0, based on the early phase of COVID-19 outbreak in Italy. Biosaf Heal. 2020;2:57–9. 10.1016/j.bsheal.2020.03.004 PMC7148916 32835209

[55] Fanelli D, Piazza F. Analysis and forecast of COVID-19 spreading in China, Italy and France. Chaos Solitons Fractals [Internet]. 2020 May;134:109761. Available from: https://linkinghub.elsevier.com/retrieve/pii/S0960077920301636 10.1016/j.chaos.2020.109761 PMC7156225 32308258

[56] Alimohamadi Y, Taghdir M, Sepandi M. Estimate of the basic reproduction number for COVID-19: a systematic review and meta-analysis. J Prev Med Public Heal [Internet]. 2020 May 31;53(3):151–7. Available from: http://jpmph.org/journal/view.php?doi=10.3961/jpmph.20.076 10.3961/jpmph.20.076 PMC7280807 32498136

[57] Lauer SA, Grantz KH, Bi Q, Jones FK, Zheng Q, Meredith HR, et al. The incubation period of coronavirus disease 2019 (COVID-19) from publicly reported confirmed cases: estimation and application. Ann Intern Med [Internet]. 2020 May 5;172(9):577–82. Available from: https://www.acpjournals.org/doi/10.7326/M20-0504 10.7326/M20-0504 PMC7081172 32150748

[58] Backer JA, Klinkenberg D, Wallinga J. Incubation period of 2019 novel coronavirus (2019- nCoV) infections among travellers from Wuhan, China, 20–28 January 2020. Euro Surveill. 2020;25(5):pii=2000062. 10.2807/1560-7917.ES.2020.25.5.2000062 PMC7014672 32046819

[59] Linton NM, Kobayashi T, Yang Y, Hayashi K, Akhmetzhanov AR, Jung S-M, et al. Clinical medicine incubation period and other epidemiological characteristics of 2019 novel coronavirus infections with right truncation: a statistical analysis of publicly available case data. mdpi.com [Internet]; 2020 [cited 2020 Aug 9];538:1–9. Available from: www.mdpi.com/journal/jcm 10.3390/jcm9020538 PMC7074197 32079150

[60] Sanche S, Lin YT, Xu C, Romero-Severson E, Hengartner N, Ke R. High contagiousness and rapid spread of severe acute respiratory syndrome coronavirus 2. Emerg Infect Dis [Internet]. 2020 Jul;26(7):1470–7. Available from: http://wwwnc.cdc.gov/eid/article/26/7/20-0282_article.htm 10.3201/eid2607.200282 PMC7323562 32255761

[61] Minskaia E, Hertzig T, Gorbalenya AE, Campanacci V, Cambillau C, Canard B, et al. Discovery of an RNA virus 3′ → 5′ exoribonuclease that is critically involved in coronavirus RNA synthesis. Proc Natl Acad Sci USA [Internet]. 2006 Mar 28 [cited 2021 Feb 11];103(13):5108–13. Available from: www.pnas.orgcgidoi10.1073pnas.0508200103 10.1073/pnas.0508200103 PMC1458802 16549795

[62] Nagy PD, Carpenter CD, Simon AE. A novel 3’-end repair mechanism in an RNA virus. Proc Natl Acad Sci [Internet]. 1997 Feb 18;94(4):1113–8. Available from: http://www.pnas.org/cgi/doi/10.1073/pnas.94.4.1113 10.1073/pnas.94.4.1113 PMC19753 9037015

[63] Chen P, Jiang M, Hu T, Liu Q, Chen XS, Guo D. Biochemical characterization of exoribonuclease encoded by SARS coronavirus. J Biochem Mol Biol [Internet]. 2007 [cited 2021 Feb 11];40(5):649–55. Available from: https://pubmed.ncbi.nlm.nih.gov/17927896/10.5483/bmbrep.2007.40.5.649 17927896

[64] Barr JN, Fearns R. How RNA viruses maintain their genome integrity. J Gen Virol [Internet]. 2010 Jun 1;91(6):1373–87. Available from: https://www.microbiologyresearch.org/content/journal/jgv/10.1099/vir.0.020818-0 10.1099/vir.0.020818-0 20335491

[65] Saunders RDC, Boubriak I, Clancy DJ, Cox LS. Identification and characterization of a Drosophila ortholog of WRN exonuclease that is required to maintain genome integrity. Aging Cell [Internet]. 2008 Jun;7(3):418–25. Available from: http://doi.wiley.com/10.1111/j.1474-9726.2008.00388.x 10.1111/j.1474-9726.2008.00388.x PMC2408639 18346216

[66] Becares M, Pascual-Iglesias A, Nogales A, Sola I, Enjuanes L, Zuñiga S. Mutagenesis of coronavirus nsp14 reveals its potential role in modulation of the innate immune response. J Virol. 2016;90(11):5399–414. 10.1128/JVI.03259-15 PMC4934755 27009949

[67] Eckerle LD, Lu X, Sperry SM, Choi L, Denison MR. High fidelity of murine hepatitis virus replication is decreased in nsp14 exoribonuclease mutants. J Virol. 2007;81(22):12135–44. 10.1128/JVI.01296-07 PMC2169014 17804504

[68] Davies M, Osborne V, Lane S, Roy D, Dhanda S, Evans A, et al. Remdesivir in treatment of COVID-19: a systematic benefit–risk assessment. Drug Saf [Internet]. 2020 Jul 28;43(7):645–56. Available from: http://link.springer.com/10.1007/s40264-020-00952-1 10.1007/s40264-020-00952-1 PMC7255634 32468196

[69] Gautret P, Lagier J-C, Parola P, Hoang VT, Meddeb L, Sevestre J, et al. Clinical and microbiological effect of a combination of hydroxychloroquine and azithromycin in 80 COVID-19 patients with at least a six-day follow up: a pilot observational study. Travel Med Infect Dis [Internet]. 2020 Mar;34:101663. Available from: https://linkinghub.elsevier.com/retrieve/pii/S1477893920301319 10.1016/j.tmaid.2020.101663 PMC7151271 32289548

[70] Zhu FC, Guan XH, Li YH, Huang JY, Jiang T, Hou LH, et al. Immunogenicity and safety of a recombinant adenovirus type-5-vectored COVID-19 vaccine in healthy adults aged 18 years or older: a randomised, double-blind, placebo-controlled, phase 2 trial. Lancet [Internet]. 2020 Aug 15 [cited 2021 Feb 11];396(10249):479–88. 10.1016/S0140-6736(20)31605-6 PMC7836858 32702299

[71] Haidere MF, Ratan ZA, Nowroz S, Zaman SB, Jung Y-J, Hosseinzadeh H, et al. COVID-19 vaccine: critical questions with complicated answers. Biomol Ther (Seoul). 2021 Jan;29(1):1–10. 10.4062/biomolther.2020.178 PMC7771841 33372165

[72] Zhu N, Zhang D, Wang W, Li X, Yang B, Song J, et al. A novel coronavirus from patients with pneumonia in China, 2019. N Engl J Med. 2020 Feb 20;382(8):727–33. 10.1056/NEJMoa2001017 PMC7092803 31978945

[73] Kuo TY, Lin MY, Coffman RL, Campbell JD, Traquina P, Lin YJ, et al. Development of CpG-adjuvanted stable prefusion SARS-CoV-2 spike antigen as a subunit vaccine against COVID-19. Sci Rep. 2020 Dec 1;10:1. 10.1038/s41598-020-77077-z PMC7676267 33208827

[74] Wang F, Kream RM, Stefano GB. An evidence based perspective on mRNA-SARScov-2 vaccine development [Internet]. Med Sci Monit. 2020 [cited 2021 Feb 11];26:1–8. Available from: https://pubmed.ncbi.nlm.nih.gov/32366816/10.12659/MSM.924700 PMC7218962 32366816

[75] Vogel AB, Kanevsky I, Che Y, Swanson KA, Muik A, Vormehr M, et al. Immunogenic BNT162b vaccines protect rhesus macaques from SARS-CoV-2. Nature [Internet]. 2021 Feb 1 [cited 2021 Feb 11];592:1–10. Available from: https://www.nature.com/articles/s41586-021-03275-y 10.1038/s41586-021-03275-y 33524990

[76] Walsh EE, Frenck RW, Falsey AR, Kitchin N, Absalon J, Gurtman A, et al. Safety and immunogenicity of two RNA-based Covid-19 vaccine candidates. N Engl J Med [Internet]. 2020 Dec 17 [cited 2021 Feb 11];383(25):2439–50. Available from: http://www.nejm.org/doi/10.1056/NEJMoa2027906 10.1056/NEJMoa2027906 PMC7583697 33053279

[77] Corbett KS, Edwards DK, Leist SR, Abiona OM, Boyoglu-Barnum S, Gillespie RA, et al. SARS-CoV-2 mRNA vaccine design enabled by prototype pathogen preparedness. Nature [Internet]. 2020 Oct 22 [cited 2021 Feb 11];586(7830):567–71. Available from: https://doi.org/10.1038/s41586-020-2622-0 10.1038/s41586-020-2622-0 PMC7581537 32756549

[78] Anderson EJ, Rouphael NG, Widge AT, Jackson LA, Roberts PC, Makhene M, et al. Safety and immunogenicity of SARS-CoV-2 mRNA-1273 vaccine in older adults. N Engl J Med [Internet]. 2020 Dec 17 [cited 2021 Feb 11];383(25):2427–38. Available from: https://pubmed.ncbi.nlm.nih.gov/32991794/10.1056/NEJMoa2028436 PMC7556339 32991794

[79] Folegatti PM, Ewer KJ, Aley PK, Angus B, Becker S, Belij-Rammerstorfer S, et al. Safety and immunogenicity of the ChAdOx1 nCoV-19 vaccine against SARS-CoV-2: a preliminary report of a phase 1/2, single-blind, randomised controlled trial. Lancet [Internet]. 2020 Aug 15 [cited 2021 Feb 11];396(10249):467–78. 10.1016/S0140-6736(20)31604-4 PMC7445431 32702298

[80] van Doremalen N, Lambe T, Spencer A, Belij-Rammerstorfer S, Purushotham JN, Port JR, et al. ChAdOx1 nCoV-19 vaccine prevents SARS-CoV-2 pneumonia in rhesus macaques. Nature [Internet]. 2020 Oct 22 [cited 2021 Feb 11];586(7830):578–82. Available from: https://doi.org/10.1038/s41586-020-2608-y 10.1038/s41586-020-2608-y PMC8436420 32731258

[81] Zhang Y, Zeng G, Pan H, Li C, Hu Y, Chu K, et al. Safety, tolerability, and immunogenicity of an inactivated SARS-CoV-2 vaccine in healthy adults aged 18–59 years: a randomised, double-blind, placebo-controlled, phase 1/2 clinical trial. Lancet Infect Dis [Internet]. 2021 Feb 1 [cited 2021 Mar 11];21(2):181–92. Available from: www.thelancet.com/infection 10.1016/S1473-3099(20)30843-4 PMC7832443 33217362

[82] Logunov DY, Dolzhikova IV, Zubkova OV, Tukhvatullin AI, Shcheblyakov DV, Dzharullaeva AS, et al. Safety and immunogenicity of an rAd26 and rAd5 vector-based heterologous prime-boost COVID-19 vaccine in two formulations: two open, non-randomised phase 1/2 studies from Russia. Lancet [Internet]. 2020 Sep 26 [cited 2021 Feb 11];396(10255):887–97. 10.1016/S0140-6736(20)31866-3 PMC7471804 32896291

[83] Lundstrom K. Viral vectors for COVID-19 vaccine development. Viruses [Internet]. 2021 Feb 19 [cited 2021 Mar 11];13(2):317. Available from: https://www.mdpi.com/1999-4915/13/2/317 10.3390/v13020317 PMC7922679 33669550

[84] Sanders B, Koldijk M, Schuitemaker H. Inactivated viral vaccines. Vaccine analysis: strategies, principles, and control [Internet]. Berlin Heidelberg: Springer; 2015 [cited 2021 Mar 11]. p. 45–80. Available from: https://link.springer.com/chapter/10.1007/978-3-662-45024-6_2

[85] Hicks DJ, Fooks AR, Johnson N. Developments in rabies vaccines [Internet]. Clin Exp Immunol. 2012 [cited 2021 Mar 11];169:199–204. Available from: https://pubmed.ncbi.nlm.nih.gov/22861358/10.1111/j.1365-2249.2012.04592.x PMC3444995 22861358

[86] Barrett PN, Mundt W, Kistner O, Howard MK. Vero cell platform in vaccine production: moving towards cell culture-based viral vaccines [Internet]. Expert Rev Vaccines. 2009 [cited 2021 Mar 11];8:607–18. Available from: https://pubmed.ncbi.nlm.nih.gov/19397417/10.1586/erv.09.19 19397417

[87] Madhusudana SN, Shamsundar R, Seetharaman S. In vitro inactivation of the rabies virus by ascorbic acid. Int J Infect Dis [Internet]. 2004 [cited 2021 Mar 11];8(1):21–5. Available from: https://pubmed.ncbi.nlm.nih.gov/14690777/10.1016/j.ijid.2003.09.002 14690777

[88] Bahnemann HG. Inactivation of viral antigens for vaccine preparation with particular reference to the application of binary ethylenimine [Internet]. Vaccine. 1990 [cited 2021 Mar 12];8:299–303. Available from: https://pubmed.ncbi.nlm.nih.gov/2204242/10.1016/0264-410X(90)90083-X PMC7173316 2204242

[89] Amanna IJ, Raué HP, Slifka MK. Development of a new hydrogen peroxide-based vaccine platform. Nat Med [Internet]. 2012 Jun [cited 2021 Mar 11];18(6):974–9. Available from: https://pubmed.ncbi.nlm.nih.gov/22635006/10.1038/nm.2763 PMC3506259 22635006

[90] Metz B, Kersten GFA, Hoogerhout P, Brugghe HF, Timmermans HAM, De Jong A, et al. Identification of formaldehyde-induced modifications in proteins: reactions with model peptides. J Biol Chem [Internet]. 2004 Feb 20 [cited 2021 Mar 12];279(8):6235–43. Available from: https://pubmed.ncbi.nlm.nih.gov/14638685/10.1074/jbc.M310752200 14638685

[91] Lawrence SA. beta-Propiolactone: viral inactivation in vaccines and plasma products. PDA J Pharm Sci Technol. 2000;54(3):209–17. 10927912

[92] Campbell CH, Barber TL, Knudsen RC, Swaney LM. Immune response of mice and sheep to bluetongue virus inactivated by gamma irradiation. Prog Clin Biol Res [Internet]. 1985 Jan 1 [cited 2021 Mar 12];178:639–47. Available from: https://europepmc.org/article/med/2989913 2989913

[93] Reitman M, Tribble HR, Green L. Gamma-irradiated Venezuelan equine encephalitis vaccines. Appl Microbiol [Internet]. 1970 [cited 2021 Mar 11];19(5):763–7. Available from: https://www.ncbi.nlm.nih.gov/pmc/articles/PMC376784/?report=abstract 10.1128/am.19.5.763-767.1970 PMC376784 5463575

[94] Wiktor TJ, Aaslestad HG, Kaplan MM. Immunogenicity of rabies virus inactivated by -propiolactone, acetylethyleneimine, and ionizing irradiation. Appl Microbiol [Internet]. 1972 [cited 2021 Mar 11];23(5):914–8. Available from: https://www.ncbi.nlm.nih.gov/pmc/articles/PMC380470/?report=abstract 10.1128/am.23.5.914-918.1972 PMC380470 5031561

[95] Marennikova SS, Macevic GR. Experimental study of the role of inactivated vaccine in two step vaccination against smallpox bull world health organ [Internet]. 1975 Jan 1 [cited 2021 Mar 11];52(1):51–6. Available from: https://www.ncbi.nlm.nih.gov/pmc/articles/pmid/1082382/?tool=EBI PMC2366348 1082382

[96] Mullbacher A, Ada GL, Tha Hla R. Gamma-irradiated influenza A virus can prime for a cross-reactive and cross-protective immune response against influenza A viruses. Immunol Cell Biol [Internet]. 1988 [cited 2021 Mar 11];66(2):153–7. Available from: https://pubmed.ncbi.nlm.nih.gov/2846435/10.1038/icb.1988.19 2846435

[97] Kang CY, Gao Y. Killed whole-HIV vaccine, employing a well established strategy for antiviral vaccines [Internet]. AIDS Res Ther. 2017 [cited 2021 Feb 11];14:47. Available from: https://aidsrestherapy.biomedcentral.com/articles/10.1186/s12981-017-0176-5 10.1186/s12981-017-0176-5 PMC5594480 28893272

[98] Marzi A, Halfmann P, Hill-Batorski L, Feldmann F, Shupert WL, Neumann G, et al. An Ebola whole-virus vaccine is protective in nonhuman primates. Science (80) [Internet]. 2015 Apr 24 [cited 2021 Feb 11];348(6233):439–42. Available from: https://pubmed.ncbi.nlm.nih.gov/25814063/10.1126/science.aaa4919 PMC4565490 25814063

[99] Shahrudin S, Chen C, David SC, Singleton EV, Davies J, Kirkwood CD, et al. Gamma-irradiated rotavirus: a possible whole virus inactivated vaccine. PLoS One [Internet]. 2018 Jun 7 [cited 2021 Feb 11];13(6):e0198182. Available from: https://dx.plos.org/10.1371/journal.pone.0198182 10.1371/journal.pone.0198182 PMC5991763 29879130

[100] Tobin GJ, Tobin JK, Gaidamakova EK, Wiggins TJ, Bushnell RV, Lee W-M, et al. A novel gamma radiation-inactivated sabin-based polio vaccine. In: Alsharifi M, editor. PLoS One [Internet]. 2020 Jan 30 [cited 2021 Feb 11];15(1):e0228006. Available from: https://dx.plos.org/10.1371/journal.pone.0228006 10.1371/journal.pone.0228006 PMC6991977 31999745

[101] Feldmann F, Shupert WL, Haddock E, Twardoski B, Feldmann H. Gamma irradiation as an effective method for inactivation of emerging viral pathogens. Am J Trop Med Hyg [Internet]. 2019 [cited 2021 Feb 11];100(5):1275–7. Available from: https://pubmed.ncbi.nlm.nih.gov/30860018/10.4269/ajtmh.18-0937 PMC6493948 30860018

[102] Jahrling PB, Stephenson EH. Protective efficacies of live attenuated and formaldehyde-inactivated Venezuelan equine encephalitis virus vaccines against aerosol challenge in hamsters. J Clin Microbiol [Internet]. 1984 [cited 2021 Mar 12];19(3):429–31. Available from: https://pubmed.ncbi.nlm.nih.gov/pmc/articles/PMC271080/?report=abstract 10.1128/jcm.19.3.429-431.1984 PMC271080 6715512

[103] Delrue I, Verzele D, Madder A, Nauwynck HJ. Inactivated virus vaccines from chemistry to prophylaxis: merits, risks and challenges. Expert Rev Vaccines [Internet]. 2012 Jun 9 [cited 2021 Mar 12];11(6):695–719. Available from: http://www.tandfonline.com/doi/full/10.1586/erv.12.38 10.1586/erv.12.38 22873127

[104] Ohshima H, Iida Y, Matsuda A, Kuwabara M. Damage induced by hydroxyl radicals generated in the hydration layer of GAMMA-irradiated frozen aqueous solution of DNA. J Radiat Res [Internet]. 1996 Sep 1 [cited 2021 Mar 12];37(3):199–207. Available from: https://academic.oup.com/jrr/article-lookup/doi/10.1269/jrr.37.199 10.1269/jrr.37.199 8996978

[105] Alsharifi M, Müllbacher A. The γ-irradiated influenza vaccine and the prospect of producing safe vaccines in general. Immunol Cell Biol. 2010;88:103–4. 10.1038/icb.2009.81 19859081

[106] Mullbacher A, Marshall ID, Ferris P. Classification of Barmah Forest virus as an alphavirus using cytotoxic T cell assays. J Gen Virol [Internet]. 1986 [cited 2021 Mar 12];67(2):295–9. Available from: https://pubmed.ncbi.nlm.nih.gov/3003237/10.1099/0022-1317-67-2-295 3003237

[107] Lowy RJ, Vavrina GA, LaBarre DD. Comparison of gamma and neutron radiation inactivation of influenza A virus. Antiviral Res [Internet]. 2001 [cited 2021 Feb 11];52(3):261–73. Available from: https://pubmed.ncbi.nlm.nih.gov/11675143/10.1016/s0166-3542(01)00169-3 11675143

[108] Patterson EI, Prince T, Anderson ER, Casas-Sanchez A, Smith SL, Cansado-Utrilla C, et al. Methods of inactivation of SARS-CoV-2 for downstream biological assays. J Infect Dis [Internet]. 2020 Oct 1 [cited 2021 Feb 11];222(9):1462–7. Available from: https://academic.oup.com/jid/article/222/9/1462/5892951 10.1093/infdis/jiaa507 PMC7529010 32798217

[109] Koopmans M, Duizer E. Foodborne viruses: an emerging problem. Int J Food Microbiol. 2004 Jan;90(1):23–41. 10.1016/S0168-1605(03)00169-7 PMC7127053 14672828

[110] Duan S-M, Zhao X-S, Wen R-F, Huang J-J, Pi G-H, Zhang S-X, et al. Stability of SARS coronavirus in human specimens and environment and its sensitivity to heating and UV irradiation. Biomed Environ Sci. 2003 Sep;16(3):246–55. 14631830

[111] Darnell MER, Subbarao K, Feinstone SM, Taylor DR. Inactivation of the coronavirus that induces severe acute respiratory syndrome, SARS-CoV. J Virol Methods [Internet]. 2004 Oct;121(1):85–91. Available from: https://linkinghub.elsevier.com/retrieve/pii/S016609340400179X 10.1016/j.jviromet.2004.06.006 PMC7112912 15350737

[112] Gerlach M, Wolff S, Ludwig S, Schäfer W, Keiner B, Roth NJ, et al. Rapid SARS-CoV-2 inactivation by commonly available chemicals on inanimate surfaces [Internet]. J Hosp Infect. 2020 [cited 2021 Feb 11];106:633–4. Available from: https://doi.org/10.1016/j.jhin.2020.09.001 10.1016/j.jhin.2020.09.001 PMC7480442 32916211

[113] Kumar A, Becker D, Adhikary A, Sevilla MD. Molecular sciences reaction of electrons with DNA: radiation damage to radiosensitization. mdpi.com [Internet]; 2019 [cited 2020 Aug 9]. Available from: www.mdpi.com/journal/ijms 10.3390/ijms20163998 PMC6720166 31426385

[114] Alizadeh E, Orlando TM, Sanche L. Biomolecular damage induced by ionizing radiation: the direct and indirect effects of low-energy electrons on DNA. Annu Rev Phys Chem [Internet]. 2015 Apr;66(1):379–98. Available from: http://www.annualreviews.org/doi/10.1146/annurev-physchem-040513-103605 10.1146/annurev-physchem-040513-103605 25580626

[115] O’Neill P, Stevens DL, Garman E. Physical and chemical considerations of damage induced in protein crystals by synchrotron radiation: a radiation chemical perspective. J Synchrotron Radiat. 2002;9(6):329–32. 10.1107/s0909049502014553 12409618

[116] Bartels DM, Cook AR, Mudaliar M, Jonah CD. Spur decay of the solvated electron in picosecond radiolysis measured with time-correlated absorption spectroscopy. J Phys Chem A [Internet]. 2000 Mar;104(8):1686–91. Available from: https://pubs.acs.org/doi/10.1021/jp992723e

[117] Uehara S, Nikjoo H. Monte Carlo simulation of water radiolysis for low-energy charged particles [Internet]. J Radiat Res. 2006 [cited 2020 Aug 9];47:69–81. Available from: http://jrr.jstage.jst.go.jp 10.1269/jrr.47.69 16571920

[118] Lytle CD, Sagripanti J-L. Predicted inactivation of viruses of relevance to biodefense by solar radiation. J Virol. 2005;79(22):14244–52. 10.1128/JVI.79.22.14244-14252.2005 PMC1280232 16254359

[119] Lomax ME, Folkes LK, O’neill P. Biological consequences of radiation-induced DNA damage: relevance to radiotherapy. Clin Oncol. 2013;25(10):578–85. 10.1016/j.clon.2013.06.007 23849504

[120] Gates KS. An overview of chemical processes that damage cellular DNA: spontaneous hydrolysis, alkylation, and reactions with radicals. Chem Res Toxicol. 2009;22(11):1747–60. 10.1021/tx900242k PMC2806061 19757819

[121] Sobotta L, Skupin-Mrugalska P, Mielcarek J, Goslinski T, Balzarini J. Photosensitizers mediated photodynamic inactivation against virus particles mini-reviews. Med Chem [Internet]. 2015 Apr 18 [cited 2021 Feb 11];15(6):503–21. Available from: https://pubmed.ncbi.nlm.nih.gov/25877599/10.2174/1389557515666150415151505 25877599

[122] Costa L, Faustino MAF, Neves MGPMS, Cunha Â, Almeida A. Photodynamic inactivation of mammalian viruses and bacteriophages [Internet]. Viruses. 2012 [cited 2021 Feb 11];4:1034–74. Available from: https://pubmed.ncbi.nlm.nih.gov/pmc/articles/PMC3407894/10.3390/v4071034 PMC3407894 22852040

[123] Lampe N, Karamitros M, Breton V, Brown JMC, Sakata D, Sarramia D, et al. Mechanistic DNA damage simulations in Geant4-DNA Part 2: electron and proton damage in a bacterial cell. Phys Med. 2018;48:146–55. 10.1016/j.ejmp.2017.12.008 29371062

[124] Sommer R, Pribil W, Appelt S, Gehringer P, Eschweiler H, Leth H, et al. Inactivation of bacteriophages in water by means of non-ionizing (UV-253.7 nm) and ionizing (gamma) radiation: a comparative approach. Water Res. 2001;35(13):3109–16. 10.1016/s0043-1354(01)00030-6 11487107

[125] de Roda Husman AM, Bijkerk P, Lodder W, van den Berg H, Pribil W, Cabaj A, et al. Calicivirus Inactivation by nonionizing (253.7-nanometer-wavelength [UV]) and ionizing (gamma) radiation. Appl Environ Microbiol [Internet]. 2004 Sep;70(9):5089–93. Available from: https://aem.asm.org/content/70/9/5089 10.1128/AEM.70.9.5089-5093.2004 PMC520909 15345386

[126] Wang T-Y, Libardo MDJ, Angeles-Boza AM, Pellois J-P. Membrane oxidation in cell delivery and cell killing applications. ACS Chem Biol [Internet]. 2017 May 19;12(5):1170–82. Available from: https://pubs.acs.org/doi/10.1021/acschembio.7b00237 10.1021/acschembio.7b00237 PMC5905413 28355059

[127] Hume AJ, Ames J, Rennick LJ, Duprex WP, Marzi A, Tonkiss J, et al. Inactivation of RNA viruses by gamma irradiation: a study on mitigating factors. Viruses. 2016;8(7):204. 10.3390/v8070204 PMC4974539 27455307

[128] Nims RW, Gauvin G, Plavsic M. Gamma irradiation of animal sera for inactivation of viruses and mollicutes–a review. Biologicals. 2011;39(6):370–7. 10.1016/j.biologicals.2011.05.003 21871817

[129] Durante M, Schulze K, Incerti S, Francis Z, Zein S, Guzmán CA. Virus irradiation and COVID-19 disease. Front Phys [Internet]. 2020 Oct 20 [cited 2021 Feb 11];8:565861. Available from: https://www.frontiersin.org/articles/10.3389/fphy.2020.565861/full

[130] Zhao L, Mi D, Hu B, Sun Y. A generalized target theory and its applications. Sci Rep [Internet]. 2015 Nov 28;5(1):14568. Available from: http://www.nature.com/articles/srep14568 10.1038/srep14568 PMC4585963 26411887

[131] Nomiya T. Discussions on target theory: past and present. J Radiat Res [Internet]. 2013 Nov;54(6):1161–3. Available from: https://academic.oup.com/jrr/article-lookup/doi/10.1093/jrr/rrt075 10.1093/jrr/rrt075 PMC3823792 23732771

[132] Pruß A, Schmidt T, Hoburg AT, Gohs U, Schumann W, Sim-Brandenburg J-W, et al. Original article originalarbeit inactivation effect of standard and fractionated electron beam irradiation on enveloped and non-enveloped viruses in a tendon transplant model. Transfus Med Hemother [Internet]. 2012 Feb [cited 2020 Aug 12];39(1):29–35. Available from: www.karger.com/tmh 10.1159/000336380 PMC3388620 22896764

[133] Feldmann F, Shupert WL, Haddock E, Twardoski B, Feldmann H. Gamma irradiation as an effective method for inactivation of emerging viral pathogens. Am J Trop Med Hyg [Internet]. 2019 May 1;100(5):1275–7. Available from: http://www.ajtmh.org/content/journals/10.4269/ajtmh.18-0937 10.4269/ajtmh.18-0937 PMC6493948 30860018

[134] Leung A, Tran K, Audet J, Lavineway S, Bastien N, Krishnan J. In vitro Inactivation of SARS-CoV-2 using gamma radiation. Appl Biosaf [Internet]. 2020 Sep 1 [cited 2021 Feb 11];25(3):157–60. Available from: https://www.liebertpub.com/doi/10.1177/1535676020934242 10.1177/1535676020934242 PMC9134622 36035758

[135] Gauvin G. RN-P Journal of Pharmaceutical Science. Gamma-irradiation of serum for the inactivation of adventitious contaminants. journal.pda.org [Internet]; 2010 Undefined [cited 2020 Aug 9]. Available from: https://journal.pda.org/content/64/5/432.short 21502047

[136] Davison AJ. Herpesvirus systematics. Vet Microbiol. 2010 Jun;143(1):52–69. 10.1016/j.vetmic.2010.02.014 PMC2995426 20346601

[137] Plavsic MZ, Daley JP, Danner DJ, Weppner DJ. Gamma irradiation of bovine sera. Dev Biol Stand. 1999;99:95–109. 10404881

[138] Moss B. Poxvirus DNA replication. cshperspectives.cshlp.org [Internet]. Vol. 5, 2013 Sep [cited 2020 Aug 14]. p. 9. Available from: http://cshperspectives.cshlp.org/

[139] Thomas FC, Davies AG, Dulac GC, Willis NG, Papp-Vid G, Girard A. Gamma ray inactivation of some animal viruses. Can J Comp Med [Internet]. 1981 [cited 2021 Feb 11];45(4):397–9. Available from: https://www.ncbi.nlm.nih.gov/pmc/articles/PMC1320171/?report=abstract PMC1320171 6802472

[140] Cox RM, Plemper RK. Structure and organization of paramyxovirus particles. Curr Opin Virol. 2017;24:105–14. 10.1016/j.coviro.2017.05.004 PMC5529233 28601688

[141] Neufeldt CJ, Cortese M, Acosta EG, Bartenschlager R. Rewiring cellular networks by members of the Flaviviridae family. naturecom [Internet]. 2018 Feb 12 [cited 2020 Aug 14];16(3):125–42. Available from: www.nature.com/nrmicro 10.1038/nrmicro.2017.170 PMC7097628 29430005

[142] House C, House JA, Yedloutschnig RJ. Inactivation of viral agents in bovine serum by gamma irradiation1. Can J Microbiol. 1990;36:737–40. 10.1139/m90-126 2123735

[143] Phan MVT, Ngo Tri T, Hong Anh P, Baker S, Kellam P, Cotten M. Identification and characterization of Coronaviridae genomes from Vietnamese bats and rats based on conserved protein domains. Virus Evol [Internet]. 2018 Jul 1 [cited 2020 Aug 14];4(2):1–12. Available from: https://www.ncbi.nlm.nih.gov/pmc/articles/PMC6295324/?report=abstract 10.1093/ve/vey035 PMC6295324 30568804

[144] Dietzgen RG, Kondo H, Goodin MM, Kurath G, Vasilakis N. The family Rhabdoviridae: mono- and bipartite negative-sense RNA viruses with diverse genome organization and common evolutionary origins [Internet]. Virus Res. 2017 [cited 2021 Feb 11];227:158–70. Available from: https://pubmed.ncbi.nlm.nih.gov/27773769/10.1016/j.virusres.2016.10.010 PMC5124403 27773769

[145] Klemm C, Reguera J, Cusack S, Zielecki F, Kochs G, Weber F. Systems to establish bunyavirus genome replication in the absence of transcription. J Virol [Internet]. 2013 Jul 15 [cited 2021 Feb 11];87(14):8205–12. Available from: http://jvi.asm.org/10.1128/JVI.00371-13 PMC3700186 23698297

[146] Akashi H, Onuma S, Nagano H, Ohta M, Fukutomi T. Detection and differentiation of Aino and Akabane Simbu serogroup bunyaviruses by nested polymerase chain reaction. Arch Virol [Internet]. 1999 [cited 2021 Feb 11];144(11):2101–9. Available from: https://pubmed.ncbi.nlm.nih.gov/10603165/10.1007/s007050050625 10603165

